# Enhanced Low-Density Silicone Foams Blown by Water–Hydroxyl Blends

**DOI:** 10.3390/polym15224425

**Published:** 2023-11-16

**Authors:** Ingrid Rebane, Karl Jakob Levin, Uno Mäeorg, Urmas Johanson, Peeter Piirimägi, Tauri Tätte, Tarmo Tamm

**Affiliations:** 1 Institute of Technology, University of Tartu, Nooruse 1, 50411 Tartu, Estonia; karl.jakob.levin@ut.ee (K.J.L.); urmas.johanson@ut.ee (U.J.);; 2 Institute of Chemistry, University of Tartu, Ravila 14a, 50411 Tartu, Estonia; uno.maeorg@ut.ee; 3 Estelaxe Ltd., Kivimurru 2, 65605 Võru, Estonia

**Keywords:** open-cell cellular structures, lightweight materials, blowing agents, silicone foams, porous PDMS, elastomeric foams, injection molding

## Abstract

Water, alcohols, diols, and glycerol are low-cost blowing agents that can be used to create the desired silicone foam structures. Although their combined use can be beneficial, it remains unclear how it affects the physical properties of the resulting materials. We conducted a comparative study of these hydroxyl-bearing blowing agents in fumed silica- and mica-filled polymer composite systems for simultaneous blowing and crosslinking to obtain a low-density, uniform porosity and superior mechanical properties. The foams were optimized for a uniform open-pore structure with densities ranging from 75 to 150 kg‧m^−3^. Varying the diol chain length (C_n_) from one to seven carbons can alter the foam density and structure, thereby enhancing the foam tensile strength while maintaining a low density. Replacing 10 mol% of water with 1,4-butanediol decreased the density by 26%, while increasing the specific strength by 5%. By combining glycerol and water blowing, the resulting foams exhibited a 30% lower apparent density than their water-blown analogs. The results further showed that C_n_ > 4 alkane chain diols had an odd–even effect on the apparent density and cell wall thickness. All foamable compositions had viscosities of approximately 7000 cSt and curing times below 2 min, allowing for quick dispensing and sufficient time for the foam to cure in semi-industrial volumes.

## 1. Introduction

Polysiloxane-based materials have become one of the most preferred materials in applications in which chemical inertness, inherent fire retardancy, and excellent physicomechanical properties are desired [1]. Their elastic ternary structures with tunable density and mechanical properties are especially attractive for medical and wearable electronics applications, as well as in the automotive and construction industries. 

Extensive research has been conducted on the preparation methods for polysiloxane-based foams, often based on two-component premixture systems (e.g., Sylgard^®^, Rhodorsil RTFoam, or Elastosil^®^ LR series) in which the exact composition is proprietary or a trade secret [2,3,4,5,6,7]. Unfortunately, to achieve a low-density rubber foam with the desired physicomechanical properties, the ready-made compositions must be significantly altered, which often have not been optimized for elastomeric foams [8]. Nevertheless, the synthesis of a silicone network, which constitutes the ternary polymer scaffold, is a relatively simple process that requires combining a selection of functionalized prepolymers, suitable crosslinking agent(s), and, depending on the reaction mechanism, a suitable catalyst [9]. By incorporating reinforcing fillers and additives, it is possible to significantly modify the strength and other mechanical properties of pristine polysiloxane elastomers, limited by the nonpolar nature of the Si-O-Si backbone and helical polymer chain [6,8]. 

Although silicone foams (SIFs) are commercially available, mainly in the form of sheets, it is important to further study the possibilities of enhancing their properties and improving the procedures used for manufacturing expandable elastomeric materials. By altering the chemical composition and, thus, tuning the reaction rates, it is possible to change the foam density, cell wall thickness, cell size, and structural homogeneity, all of which eventually directly affect the mechanical properties of the foam [10,11,12]. 

As low-density foams are often desirable, numerous physical and chemical blowing methods have been applied, of which saturation with supercritical CO_2_ [13] and sacrificial templating (citric acid [14], NaCl [5], sugar [15,16,17], and water [18]) have been reported to achieve porous end results. In addition, by generating oxygen as a by-product, simultaneous foaming during crosslinking has been reported by Yan et al. [19]. Often, these methods restrict the production of foams in large quantities and yield inhomogeneous morphologies. Using either chemical or physical blowing or their combination, the timing of crosslinking to capture the evolving gas is another aspect that needs to be considered [20]. 

In addition to inherent blowing by dehydrocondensation between silanol-functionalized (OH-PDMS) and hydrogen-functionalized (PMHS) silicones to propagate expansion, blowing agents are typically used [11,21,22]. The high Si-H bond reactivity allows thermodynamically highly favored nucleophilic substitution by water, alcohols, or alkoxysilanes [23]. 

**Figure 1:**
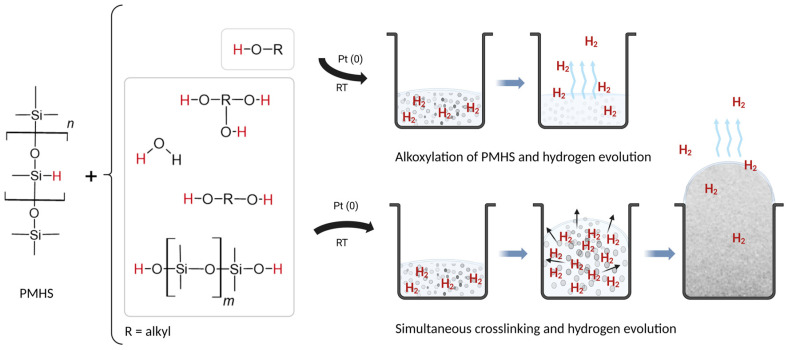
Alkanols, as blowing agents, simultaneously raise and crosslink the polymer mixture. Monoalcohols, as an exception, contribute to foam rise but do not act as crosslinkers. The silanol-terminated PDMS reacts with PMHS similarly, although as a high molecular weight polymer has less impact on the crosslinking.

Water is not an uncommon additive in silicone foams; it has been applied as a liquefier to minimize the split and tear during foam expansion [24], as a pore-former in water–silicone emulsions [25,26], and as an additional or primary blowing agent [2,3,27,28]. Recent studies have applied water as a blowing agent in commercially available prepolymer mixtures and described its softening effect on foams [3]. Monoalcohols (e.g., ethanol and isopropyl alcohol) are standard blowing agents in the literature and have been described as effective additives [2,29]. Although monoalcohols, which have only one reaction function, do not form crosslinks, each alcohol molecule contributes to foam rise by donating a hydrogen atom to the evolving H_2_ (see Figure 1).

A recent report showed the use of isopropyl alcohol and water on a silicone foam’s microstructure based on a commercial two-part premix, although the foam had closed cells and the densities were not discussed [2]. Conversely, dialcohols (diols) can form crosslinks, the rigidity of which can potentially be reduced by increasing the length of the alkane chain between the two reaction sites. The additional hydroxyl groups in glycerol molecules (propane-1,2,3-triol) can contribute significantly to the blowing, but their performance depends strongly on the steric accessibility to the hydroxyl groups [30]. Previously, a combination of ethanol in glycerol has been shown to produce medium-density foams, whereby increasing the ethanol content in glycerol (0.5–1.1%) and an increase in the glycerol/ethanol mixture content, in general, lowers the relative density of the foam by approximately 15–20%, remaining near 300 kg‧m^−3^ [31,32]. There have only been a handful of reports on the use of water–alkanol, especially water–glycerol mixtures, but their application in morphology enhancement is gaining more interest in continuous processes using injection molding. A comparison of silicone foams and their physicomechanical properties found in recent studies is listed in Table A1 in Appendix A.

**Figure 2:**
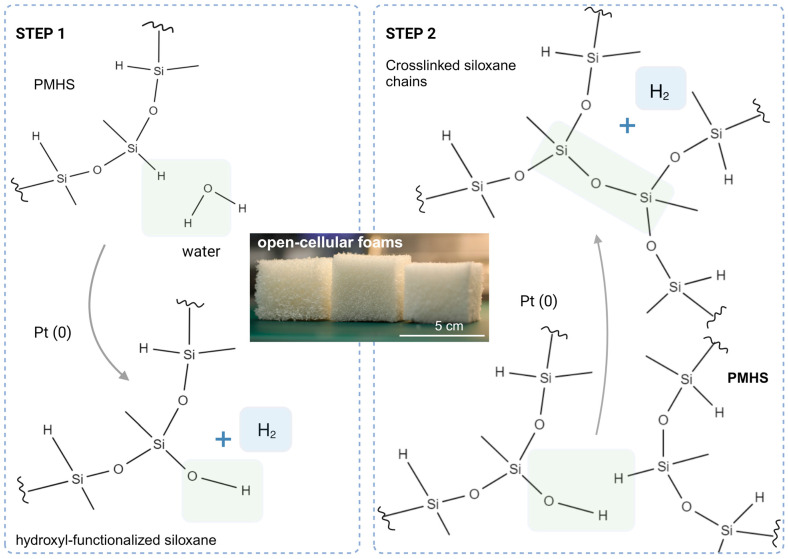
The catalyzed dehydrocondensation between water and poly(methylhydrosiloxane) (PMHS) is a two-step process, resulting in crosslinks between the PMHS chains and a porous structure that is enhanced by the evolution of hydrogen during both steps (step 1 and step 2).

This study aimed to design a curable silicone polymer base to withstand the expansion of the foam during synthesis and injection molding, allowing for the achievement of low-density polysiloxane foams (SIFs), preferably ≤100 kg‧m^−3^. The tunability of the desired physicomechanical properties is essential for such foams to suit various applications. While water as a blowing and crosslinking agent (Figure 2) has generally shown promising results in terms of the apparent density and homogeneity of foams [27], it is somewhat lacking in creating optimal mechanical properties. Therefore, one option is to partially replace water with mono-, di-, or polyalcohols, allowing for the tuning of the rates of both addition and dehydrocondensation reactions.

In our study, SIFs were obtained from a combination of polysiloxane prepolymers, crosslinked, and expanded at room temperature in the presence of a platinum catalyst (Pt(0)) and injection molded in a continuous process. The elastomer network of SIFs was strengthened by incorporating fumed silica (FS) and muscovite mica into the premixtures, both of which have been used in silicone elastomers and have been shown to enhance their physicomechanical properties and thermal stability [31]. In this designed silicone base, we varied the mole percentages (mol%) of the mono- and dialcohol solutions in water, which were applied as additional blowing additives. Several combinations showed a significant strengthening effect on the foam, resulting from alterations in the crosslinking and dehydrocondensation reaction kinetics.

## 2. Materials and Methods

### 2.1. Preparation of Polysiloxane Elastomer Foams

All foams used in the physicomechanical analysis were prepared by combining vinyl- and hydroxyl-functionalized poly(dimethylsiloxane) (5k cSt), poly(methyl hydro)siloxane (100% H, 25–35 cSt), platinum(0)-1,3-divinyl-1,1,3,3-tetramethyldisiloxane complex (Karstedt’s catalyst) obtained from Hubei Chem, moderator (2,4,6,8-tetramethyl-2,4,6,8-tetravinylcyclotetrasiloxane), and general strengthening fillers: hexamethyldisilazane (HMDS)-treated fumed silica from Gelest Inc. (Morrisville, PA, USA) and muscovite mica (KAl_2_(Si_3_Al)O_10_(OH)_2_) from OMYA, Elnesvågen, Norway. 

Methanol (Sigma-Aldrich, St. Louis, MO, USA), ethane-1,2-diol (Sigma-Aldrich, 99%), and propane-1,2-diol (Fluka Honeywell, distributor HNK Analüüsitehnika OÜ, Estonia, pur.), propane-1,3-diol (Ferak, Berlin, Germany, 96%), propane-1,2,3-triol (Lah-ner, Cambridge, MA, USA), butane-1,4-diol (Acros Organics, Morris Plains, NJ, USA), pentane-1,5-diol (Fluka, puriss., GC grade), and hexane-1,6-diol (Fluka, pur.), and heptane-1,7-diol (Fluka, pur.) were dried on molecular sieves before use.

For the prepolymer mixtures, we used a standalone mixer with a PTFE-covered rotary blade. In this study, the SIF matrix comprised OH-PDMS (100 parts), V-PDMS (50 parts), PMHS (20 parts), fumed silica (20 parts), and mica (5 parts). Karstedt’s catalyst Pt(0) (25 ppm) and four equivalents of moderator were added. The additional blowing blend, either water or a water–hydroxyl blend, was combined with the prepolymer during mixing. Next, the premixtures were combined and injected using an in-house injection molding device, dispensing a volume of 500 mL of premixture per foam sample.

Changes in the composition were made by adjusting the mole percentage (shown as mol%) based on the reactive hydroxyl groups (-OH) in alkanols and water (H-OH). We considered the water molecule to consume two equivalents of silicon hydride, reacting stepwise: initially, a dehydrocondensation reaction involving Si-H and HO(R-)OH groups, followed by a possible dehydrocondensation reaction with the formed silanol (Si-OH) and another available hydride (Si-H) in the vicinity (Figure 2).

### 2.2. Determining the Physicomechanical Properties of the Prepared Foams

We used the pycnometry technique to measure apparent density (kg‧m^−3^). The expanded measurement uncertainty was U(density, molded) = 3 kg‧m^−3^ with a confidence interval of 95% (k = 1.9), which showed the excellent reproducibility of the foam using the injection molding technique.

We measured the elongation and tensile strength of the foam samples using a motorized test stand (model AEL-A-1000, 3–1000 N range, and 300 mm‧min^−1^ test speed) following the procedures in the ISO 1798:2008 [33] and ASTM D 3574-17 [34] standards. The test specimens were die-cut from a flat sheet of material with the foam rising in the thickness direction and were free of ragged edges. The foam specimens were secured using a screw-type jagged plate grip that exerted uniform pressure across the gripping surface. Before the mechanical measurements, the samples were thoroughly post-cured in an oven at 80 °C for at least 2 h. All measurements were performed in triplicate, and the expanded measurement uncertainty for each foam sample was calculated.

The morphology of the foam was determined using SEM (TM-3000, Hitachi High-Technologies Corporation, Tokyo, Japan) at 15 kV, for which the cubical samples were sputter-coated with a 10 nm thick gold layer (Leica EM ACE600 Sputter Coater, Wetzlar, Germany). We measured the average cell diameter and cell wall thickness of each foam from the cross-section of the torn test samples using the Fiji application in ImageJ 1.54f [35]. The results are expressed as the average of 100 measurements for each sample.

Infrared spectra were collected using FTIR (Bruker, Billerica, MA, USA, Platinum-ATR, diamond crystal) to characterize the structure of the silicone foams, uncured mixture, and PMHS. The spectra can be found in the Supplementary Files.

## 3. Results and Discussion

### 3.1. Reaction Mechanisms of SIF Expansion

The foam synthesis experiments revealed that varying the mole percentages of the water and alkanol as blowing and crosslinking agents allowed for distinct changes in the microstructure and mechanical properties of the foam. Some of these effects are described and evaluated in this section to outline a clear path for the preparation of silicone foams with the desired morphology and mechanical properties. The use of water as the blowing agent in our composition resulted in low-density foams with a uniform structure. Despite this considerable advantage, the mechanical properties of such foams depend on the relatively short and stiff crosslinks, which may result in a mechanically weak and brittle polymer scaffold. Although water is not an alkanol, it contributes to dehydrogenation with both H-O bonds in a two-step process, converting the Si-H on the PMHS chain to a Si-OH and releasing hydrogen as a side product (step 1, Figure 2). The formed Si-OH groups readily react with the closest unreacted Si-H bond in the vicinal PMHS chain in the presence of a catalyst, producing additional hydrogen and forming a crosslink between siloxane chains (step 2, Figure 2) [36].

As the length of the formed crosslinks and the crosslink density affect the mechanical properties of the polymer network, dialcohols (diols), which are the shortest alkanol analogs of water, can be considered as candidates for tuning the physicomechanical properties of the foam. Similarly, via the dehydrocondensation reaction, diols contribute to strengthening the polymer material but also influence the apparent density of the resulting foam.

### 3.2. Tuning Structure and Mechanical Properties of Foams with Diols

**Figure 3:**
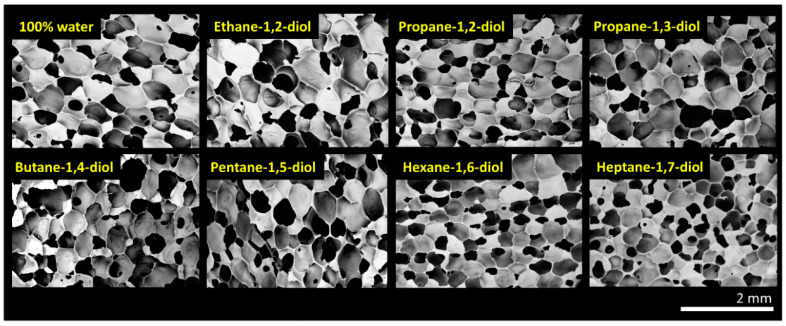
Comparison of the morphologies of foams blown with 10 mol% diols in water and 100 mol% water. Compared to water-only blowing, the 10 mol% replacement of water with diols caused slight structural changes in the foam morphology.

The use of water as the reactive foam blowing additive produced low-density foams with uniform structures. To increase the tensile strength at low densities, water was partially replaced with diol. We were interested in industrially feasible dialcohols ranging from ethane-1,2-diol (ethylene glycol) to heptane-1,7-diol. We tested diol contents ranging from 5 mol% to 100 mol% in the primary blowing composition. However, increasing the diol/water ratio increases the diol volume fraction in the premixture, causing a significant decrease in viscosity. In addition, because the accompanying increase in the exothermic effect of the dehydrocondensation reaction is undesirable, we chose to use 10 mol% in this comparison. Depending on the blowing blend used, the viscosities of the polymer mixture components remained at approximately 7000 ± 200 cSt. By partially replacing water with a diol and considering the reaction stoichiometry and molarity, a change in the foam structure was immediately observed (Figure 3).

**Figure 4:**
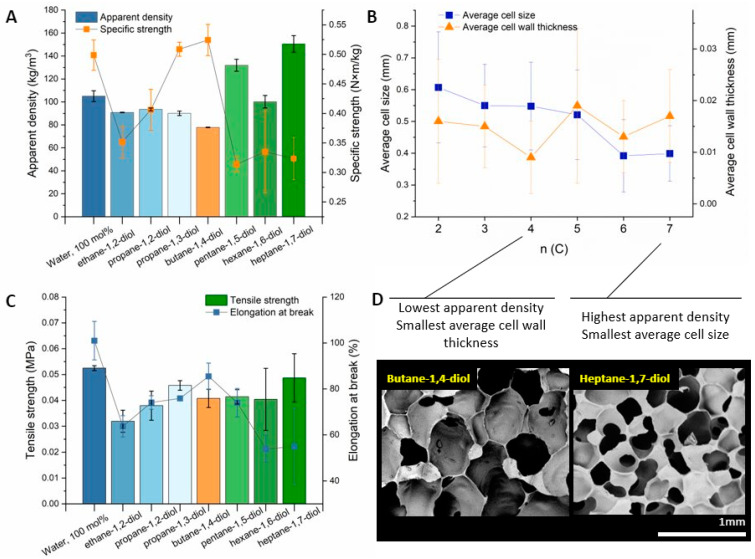
Changes in the foam density and structure for 100 mol% water-only blown foams compared to foams blown with 10 mol% diol solutions in water (**A**). The respective changes in elongation at break, tensile strength, and specific strength dependent on the apparent density are depicted in images (**A**,**C**). A general trend in cell size decrease is observed along with an increase in the chain length of the diol (**B**,**D**).

The alkane chain length C_n_ from n = 2 to n = 7 does not correlate linearly with the apparent density (Figure 4A,B), which was similar for ethane- and both propane diols; we achieved the minimum density value with 10 mol% butane-1,4-diol. An increase in the density in the order C_4_ < C_6_ < C_5_ < C_7_ could also be explained by the odd–even effect seen in alkanes [37]. The odd-numbered alkanes (n = 5, 7, and 9) exhibit up to 30 times slower dynamics than the even-numbered alkanes near their melting points. Because the molecular structure of a diol consists of an alkane chain, such an analogy could be applied to diols. This could explain the resulting higher densities of foams with propane-1,3-diol, pentane-1,5-diol, and heptane-1,7-diol and the lower densities for those with an even number of carbons in the alkane chain (n = 4 and 6) (Figure 4).

Assuming that slower dynamics affect crosslinking in the polymer mixture, the evolving gas gathers and is more likely to escape, allowing the gas bubbles to coalesce with the thickening of the pore walls. It has previously been reported that rheological properties, such as the viscosity of the mixture, affect cell growth and control bubble coalescence during crosslinking [9,38].

In addition, the experimental data based on the foam morphology (Table A2 in the Appendix A) suggest that the increase in apparent density has a strong correlation with the average wall thickness. The average cell size for foams prepared with 10 mol% diol substitution in water showed a clear decreasing trend with the increasing chain length (C_n_). Interestingly, the cell wall thickness correlated with the apparent density of the foams, supporting the odd–even effect proposed earlier.

As seen from the tensile strength measurements (Figure 4C), the foams with 10 mol% propane-1,3-diol and butane-1,4-diol exhibited the highest specific strengths (N‧m‧kg^−1^) at the lowest apparent densities (Figure 4A). Interestingly, with 10 mol% butane-1,4-diol, the resulting cell wall was thinner than that of the foams blown with 10 mol% propane-1,3-diol, although the average cell sizes were similar. In conclusion, butane-1,4-diol was the most effective crosslinking blowing agent in the 10 mol% solutions for producing lightweight, firm foams with the highest specific strength among the diols, surpassing the water-only foams. The tensile strength of the water-only foams remained the highest, next to the other 10 mol% diol–water blown foams, and can be beneficial in applications in which this property is more important than the apparent density.

### 3.3. Tuning of Foam Morphology with Methanol

**Figure 5:**
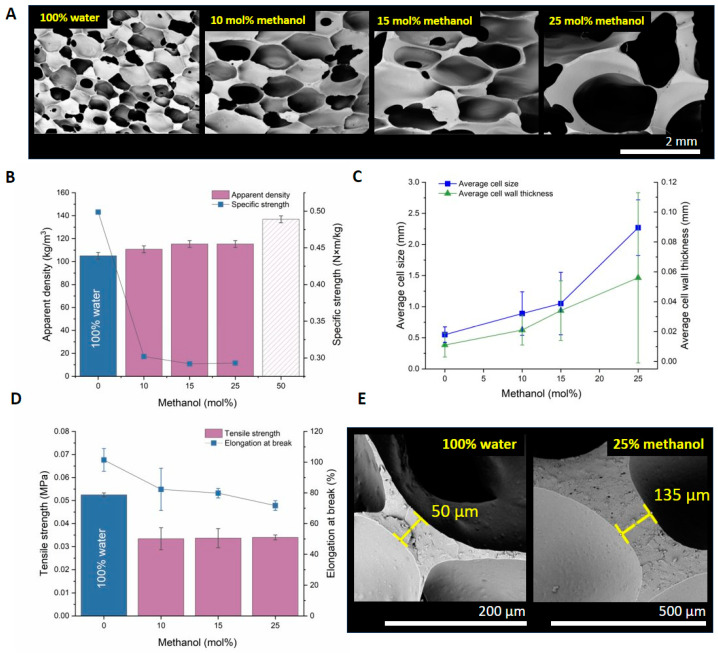
(**A**) Effect of increasing the methanol mol% in water on the morphology of the cured foam. (**B**) A higher methanol/water ratio resulted in higher densities, partially due to the decreased number of crosslinks formed and (**C**,**E**) increase in cell size and wall thickness. (**D**) Both the tensile strength and elongation at break remained relatively constant despite structural changes (**A**,**C**).

Although using water or water–diol combinations was successful in terms of the apparent density and pore uniformity, the obtained material exhibited a memory effect and a lack of stiffness under load, which is assumed to be a consequence of the small pore sizes and an increased ratio of the crosslinks. One option is to tune the rigidity of the foam by increasing its pore size and pore wall thickness [39]. This could be achieved by using monoalcohols, neither significantly altering the composition of the prepolymer mixture nor the amount of evolved hydrogen. We intended to suppress both the crosslinking and hydrogen evolution, allowing for the formation of larger voids. However, methanol has shown rapid Si-H conversion in dehydrocondensation reactions with PMHS in previous research [40]. By varying the mole percentage (mol%) of the methanol OH groups in the alcohol–water mixtures, we observed changes in the morphology, apparent density, and tensile strength. In general, an increase in the ratio of methanol (and the respective OH groups) was accompanied by a significant increase in the pore size (Figure 5A,C,E).

Methanol does not form crosslinks between siloxane chains; therefore, a lower crosslinking degree supports a slower polymer mixture solidification. We suggest that the steric hindrance of the increasing number of -OCH_3_ functionalized chains retards the overall crosslinking process by restricting the access to the -H sites on the PMHS chain. Our previous research found that the dehydrogenative coupling reaction of PMHS with methanol is rapid during the initial stage but gradually slows down, allowing for the slower evolution and convolution of hydrogen [40]. The resulting crosslinking degree decreased with an increase in the mole percentage of methanol in water while maintaining a constant number of -OH groups. This way, smaller gas-filled voids have time to coalesce, allowing for the formation of larger voids in the solidifying polymer mixture [39]. As depicted in Figure 5B,C, the increase in the methanol/water ratio caused an increase in both the cell wall thickness and cell diameter, accompanied by an increase in the apparent density of the foam. Similarly, Tan et al. showed that increasing the content of ethanol as a single blowing agent has a similar effect on foam morphology [29]. In our experiments, we kept the -OH/-H stoichiometric ratio in methanol/water mixtures constant, so changes in viscosity and crosslink density, and not the change in hydrogen volume, were the key aspects that affected the resulting structure. 

Based on these measurements, the tensile strength did not change significantly in the 10–25 mol% range of methanol in water mixtures (see Figure 5D). This range corresponds to apparent densities of 108–115 kg‧m^−3^. A further increase in the methanol content caused a significant change in the structure of the foam. The increase in the average wall thickness and pore diameter accompanied lower elongation at break values, which may result from a higher occurrence of defects, leading to premature breakage of the test sample. For foams with 50 mol% methanol, the ratio of the pore diameter and the test piece cross-sectional area did not allow for consistent tensile test measurements (see Figure 5C and cell wall and diameter data in Table A3 in Appendix A).

Consequently, replacing 10 mol% of water with methanol was sufficient to maintain a low density (110 kg‧m^−3^, +5% compared to 100% water-blown foam) with an altered morphology. Nevertheless, the tensile strength and elongation at break values were significantly lower (decreases of nearly 20% and 40 %, respectively). Loss in mechanical properties is not the expected advancement, although such foams could offer increased compression strength from a morphological aspect.

### 3.4. Optimizing Tensile Strength and Density with Diols

**Figure 6:**
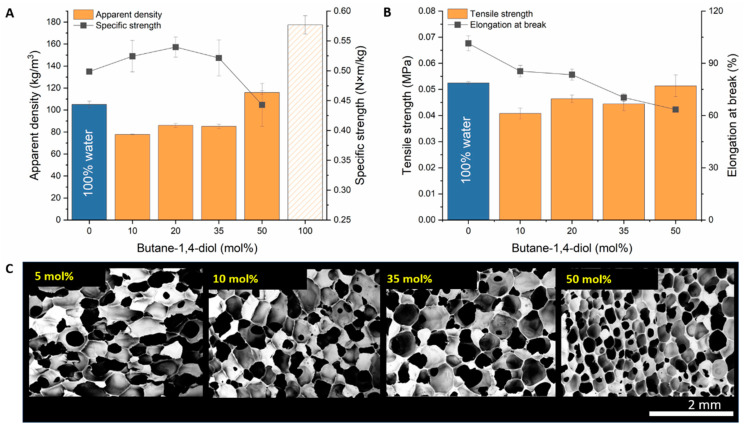
Comparison of the apparent density (**A**) and tensile strength (**B**) of foams synthesized with water and butane-1,4-diol/water solutions. The specific strength near the apparent density of 80 kg‧m^−3^ is relatively high, particularly for the foam with 10 mol% butane-1,4-diol. Increasing the butane-1,4-diol above 35mol% in water changes the cell diameter and cell wall thicknesses drastically (**C**).

When increasing the mol% of a specific diol in water, for example, butane-1,4-diol from 5 to 50 mol%, we can see a general increase in the apparent density and tensile strength and a decrease in elongation at break (Figure 6A,B).

The lowest densities were reached for 5 and 10 mol% butane-1,4-diol, but concentrations above 50 mol% had clearly undesirable density-increasing effects (>150 kg‧m^−3^). Although there is no significant difference in the elongation at break from 5 to 20 mol%, the general trend seems to be the loss in elongation together with the density increase. The average pore diameter did not significantly change when the butane-1,4-diol content was increased from 5 mol% to 35 mol%; the dominant effect for density increase was the increase in cell wall thickness near and above 50 mol% butane-1,4-diol solutions (Figure 6C).

### 3.5. Comparison of Water and Water–Diol Blown Foams

Considering the density of the foam, the specific strength is relatively high near the apparent density of 80 kg‧m^−3^, especially for foams with 10 mol% of butane-1,4-diol (0.52 N‧m‧kg^−1^, combined uncertainty U_c_ = 0.07), being comparable with 100% water-blown foam (density = 100 kg‧m^−3^ and specific strength 0.55 (U_c_ = 0.02) N‧m‧kg^−1^) (Figure 6A). Relatively good results were also obtained with 20 mol% butane-1,4-diol blown foam, which still had a lower apparent density than 100% water-blown foams; in addition, the tensile strength was the highest among foams with a density of <90 kg‧m^−3^. Hence, it had a considerably high specific strength value of 0.54 (U_c_ = 0.05) N‧m‧kg^−1^.

We did not consider a further increase in the diol content for the reasons mentioned above, and to increase the viscosity during the reaction, we would have needed to alter the main composition of the premix and adjust the catalyst concentration, making the results incomparable. Maintaining a reasonable viscosity is crucial for trapping the evolving gas during foam expansion and elastomer curing processes. We also tested the 100 mol% butane-1,4-diol blown foam but could not perform tensile strength measurements because of its brittle and weak structure. In addition, the resulting foam density is undesirable (>120 kg‧m^−3^).

### 3.6. Effect of Functional Group Position

**Figure 7:**
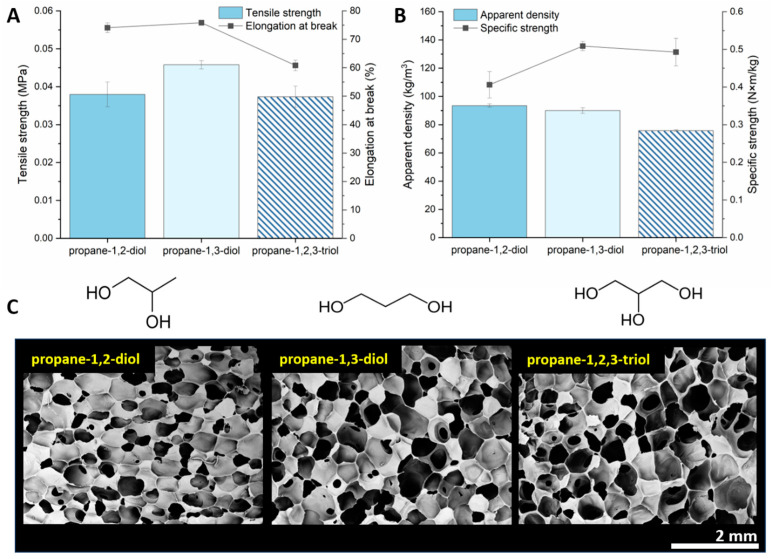
(**A**) Potentially reactive hydroxyl groups in propanols with distinct steric hindrance (accessibility of the reaction site in the respective molecule) and their effect on the tensile strength and elongation at break (**A**), specific strength (**B**), and apparent density (**C**). The importance of the OH groups in di- or triols lies in their ability to react with the PMHS chain and form a crosslink, adding or decreasing the flexibility of the crosslinked molecular structure.

When the location of the hydroxyl group in the alkane chain is varied, the access to reactive sites also changes. For propane-1,3-diol, there is less steric hindrance in the linear chain end-capped with hydroxyl groups compared to the vicinal hydroxyl groups in propane-1,2-diol, which may encounter hindered reactivity following the dehydrocondensation reaction with the first available reactive site [41]. For glycerol (propane-1,2,3-triol), the steric hindrance at carbons C_1_ and C_3_ is lower than that at the second carbon, C_2_. Depending on the polymer chain entanglement and vicinity of the PMHS chain and catalyst, the -OH attached to C_2_ is also prone to react, resulting in an additional bond that may cause the rigidity of the crosslinked material, thus altering the elasticity of the foam (lower elongation at break and lower tensile strength) (Figure 7).

Nevertheless, foams blown and simultaneously crosslinked with 10 mol% glycerol in water have an apparent density of 76 kg‧m^−3^, which is approximately 30% lower than that of the water-only blown foam. Perhaps the lower density is unsurprising since there are initially three hydroxyl groups in each glycerol molecule that form crosslinks with the Si-H bond. With each crosslink formation, the viscosity of the reaction mixture increased, thus preventing the evolution of gas from escaping. In addition, Nakagawa et al. reported that the vicinal OH groups in triols, compared to monoalcohols attached to polymer chains, show significant retardation in chain dynamics [30]. It is important to stress that the R(-OH)/PM(-H)S ratio remained constant for all experiments, so the resulting moles of hydrogen would remain constant.

## 4. Conclusions

This study used water and its blends with methanol, glycerol, and dialcohols as additional blowing agents to expand fumed silica- and mica-reinforced polysiloxane elastomer networks during crosslinking. Owing to the dehydrogenative coupling mechanism, the water–hydroxyl blends offer additional crosslinking options for polysiloxane chains. Varying the molar ratios, structure of the diols, and alkane chain length allows for the physicomechanical properties and morphology of the foam to be easily tuned. 

When water was partially substituted with a diol (10 mol%), the apparent density and tensile strength both gradually decreased from C_2_ to C_4_. From C_4_ and above, the odd–even effect for alkane chains seems to describe the correlation between the diol chain length and the alternating values of the apparent density and cell wall thickness. Compared to the 100% water-blown foam, replacing 10 mol% with butane-1,4-diol resulted in the lowest density among all the diols, whereas the specific strength values remained comparable to that of the water-only blown foam. Further increasing the diol content caused the apparent density and tensile strength to increase. The dominant effect on the density increase is the increase in cell wall thickness near and above 50 mol% butane-1,4-diol solutions.

Compared to triols, diols allow for the fabrication of foams with higher elongation at break values, and dispensing and molding are more effective owing to the reasonable viscosity of the curing and expanding mixture. A similar conclusion can be drawn for the water-only blown foams. We find this aspect to be especially important for industrial production, where small cavities need to be filled. For triols, higher ratios may also result in decreased elongation at break values because of the increased number of relatively short and closely positioned crosslinks. 

Methanol, as a monoalcohol, was introduced to the blowing blend to decrease the crosslinking degree while maintaining the amount of evolving gas. The resulting changes in the foam structure demonstrate the tunability of the foam’s physicomechanical properties, including bypassing the memory effect in water-only blown foams, and they can be implemented in various cushioning applications, in which the density increase arising from the wall thickness and cell size increase is not an issue.

Chemical blowing in the form of inherent gas-generating reactions yields the most consistent, controllable, and repeatable results regarding the apparent density, pore structure, and mechanical properties. Finding a balanced formula in this multireaction system for a desired application is an ongoing process; however, our results show that the synthesis of moldable low-density elastomeric silicone foams with desired properties is achievable.

## Data Availability

The data that support the findings of this study are available in the Supplementary Materials of this article and from the corresponding author upon reasonable request.

## Supplementary Materials

The following supporting information can be downloaded at: https://www.mdpi.com/article/10.3390/polym15224425/s1, Figure S1: IR spectrum of a water-blown foam; Figure S2: FTIR spectra of uncured polymer blend with water; Figure S3: IR spectrum of a PMHS polymer; Figure S4: IR spectra comparison of PMHS, uncured blend, and cured foam; Figure S5: IR spectra comparison of SIFs.

## Appendix A

**Table A1:** Comparative data on the physicomechanical properties of silicone-based foams produced by applying additional blowing agents or techniques.

Polymer System	Blowing Agent	Density, (kg‧m^−3^)	Elongation at Break (%), E_b_	Tensile Strength, (kPa)	Average Cell Size, (mm)	Average Wall Thickness, (mm)	Comments	Curing	Reference
**Water**									
Vi-PDMS/OH-PDMS/PMHS	water	220	-	-	0.200–0.720	0.030–0.060	surfactant for W/O emulsion	RT (several minutes)	[26]
Vi-PDMS/OH-PDMS/PMHS	water (0.2–1.8 wt%)	214	-	-	0.510	-	Inhibitor used	15 min at RT + 2 h at 100 °C	[28]
Vi-PDMS/OH-PDMS/PMHS	water (1 wt%)	105	101	52.5	0.6, SD = 0.2	0.011, SD = 0.008	-	3 min at RT	This work
PDMS/CFPS/D-66	water (0.07 mL, 1.33%)	250	42	35	>1	-	isocyanate + water reaction	RT (3–5+ min)	[27]
PDMS/CFPS/D-17	water (0.07 mL, 1.33%)	240	-	-	>1	-	isocyanate + water reaction	RT (3–5+ min)	[27]
PDMS/PMDI	water (0.07 mL, 1.12%), CO_2_	270	-	-	>1, 0.4–1.0	-	isocyanate + water reaction	RT (3–5+ min)	[27]
Elastosil LR 3003/50	water (1–3 phr)	−52%	-	-	0.005 (mm^2^)	n/a	water mixed with silica (8:1)	140 °C/180 °C + 4 h/200 °C	[3]
**Alcohols**									
Vi-PDMS/H-PDMS/PMHS	ethanol (0 wt%)	450	73	325.6	0.55	0.95		3 h at 80 °C	[29]
Vi-PDMS/H-PDMS/PMHS	ethanol (1.5 wt%)	200	31	52.8	1.6	1.4		3 h at 80 °C	[29]
Vi-PDMS/OH-PDMS/PMHS	1:9 methanol/water (1 wt%)	111	84	41.8	0.9, SD = 0.4	0.021, SD = 0.010		3 min at RT	This work
Vi-PDMS/OH-PDMS/PMHS	1:9 butane-1,4-diol/water (1 wt%)	78	86	40.8	0.6, SD = 0.2	0.009, SD = 0.007		3 min at RT	This work
**Other**									
OH-PDMS/PMHS (Rhodorsil RTFoam 3240)	no additional blowing agent, H_2_ from reaction	197	-	-	0.486 +/− 0.006	-		RT	[4]
VMQ/DBPMH/MHS	scCO_2_	109–548	-	-	0.073–0.291	0.019	compounded rubber sheets + scCO_2_	50 °C to 80 °C	[42]
**Combinative**									
Sylgard 184	glycerol (137 phr)/ethanol (1 wt%)	282	-	-	1.37 ^a^	-		120 °C	[32]
Sylgard 184	glycerol (110 phr)/ethanol (1.1 wt%)	300	-	-	1.63 ^a^	-		120 °C	[32]
Vi-PDMS/OH-PDMS/PMHS	10 mol% glycerol/water (1 wt%)	76	61	37.3	0.632, SD = 0.141	0.016, SD = 0.010		3 min at RT	This work

^a^ Values derived from foam cell size (mm^2^) data for comparison (Mazurek et al 2019).

**Table A2:** Average cell diameter, cell wall thickness, and apparent density of foams with different mol% of diols in water with varying alkane chain lengths and structures (i.e., propane-1,2-diol). SD is the standard deviation of the mean of 100 measurements.

R-(OH)*_n_*	Diol, (mol%)	Water, (mol%)	Average Wall Thickness, (mm)	Average Cell Size, (mm)	Apparent Density, (kg‧m^−3^) (*U* = 3 kg‧m^−3^)
Water	0	100	0.011, SD = 0.008	0.6, SD = 0.2	105
Ethane-1,2-diol	10	90	0.016, SD = 0.012	0.6, SD = 0.2	91
Propane-1,2-diol	10	90	0.013, SD = 0.008	0.6, SD = 0.2	94
Propane-1,3-diol	10	90	0.015, SD = 0.008	0.6, SD = 0.2	90
Butane-1,4-diol	10	90	0.009, SD = 0.007	0.6, SD = 0.2	78
Pentane-1,5-diol	10	90	0.019, SD = 0.015	0.5, SD = 0.2	132
Hexane-1,6-diol	10	90	0.013, SD = 0.007	0.4, SD = 0.2	100
Heptane-1,7-diol	10	90	0.017, SD = 0.009	0.4, SD = 0.1	151

**Table A3:** Structural parameters for foams with increasing methanol content; SD is the standard deviation of 100 measurements.

R-(OH)n	Alkanol, (mol%)	Water, (mol%)	Average Wall Thickness, (mm)	Average Cell Size, (mm)	Apparent Density, (kg‧m−3) (U = 3 kg‧m−3)
Methanol	0	100	0.011, SD = 0.008	0.6, SD = 0.2	105
	10	90	0.021, SD = 0.010	0.9, SD = 0.4	111
	15	85	0.034, SD = 0.020	1.1, SD = 0.5	115
	25	75	0.056, SD = 0.056	2.3, SD = 0.5	115
