# Supplementary figures and images for: Enhanced Low-Density Silicone Foams Blown by Water–Hydroxyl Blends
Source: Polymers (Basel). 2023 Nov 16;15(22):4425. doi: 10.3390/polym15224425 (PMC10675139; doi:10.3390/polym15224425)

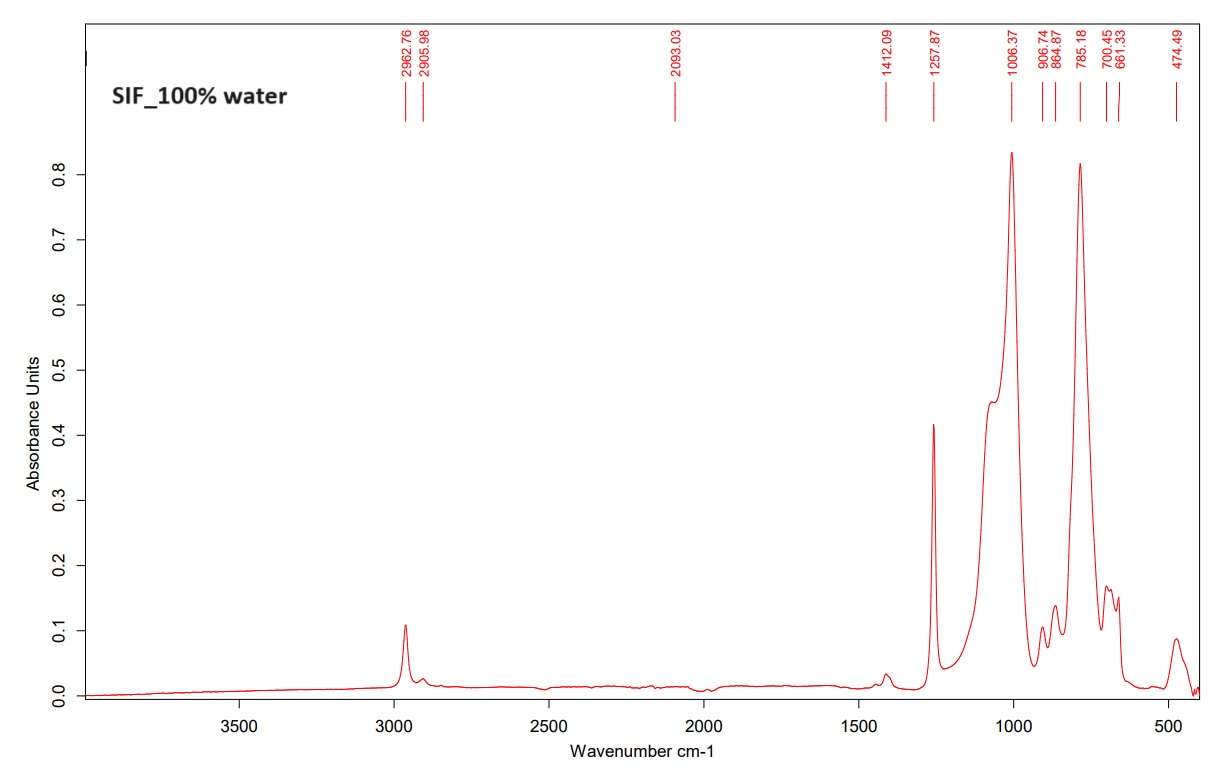

Supplement: Supplementary file 1 [file polymers-15-04425-s001.zip › Figure S1_FTIR_100water.jpg]

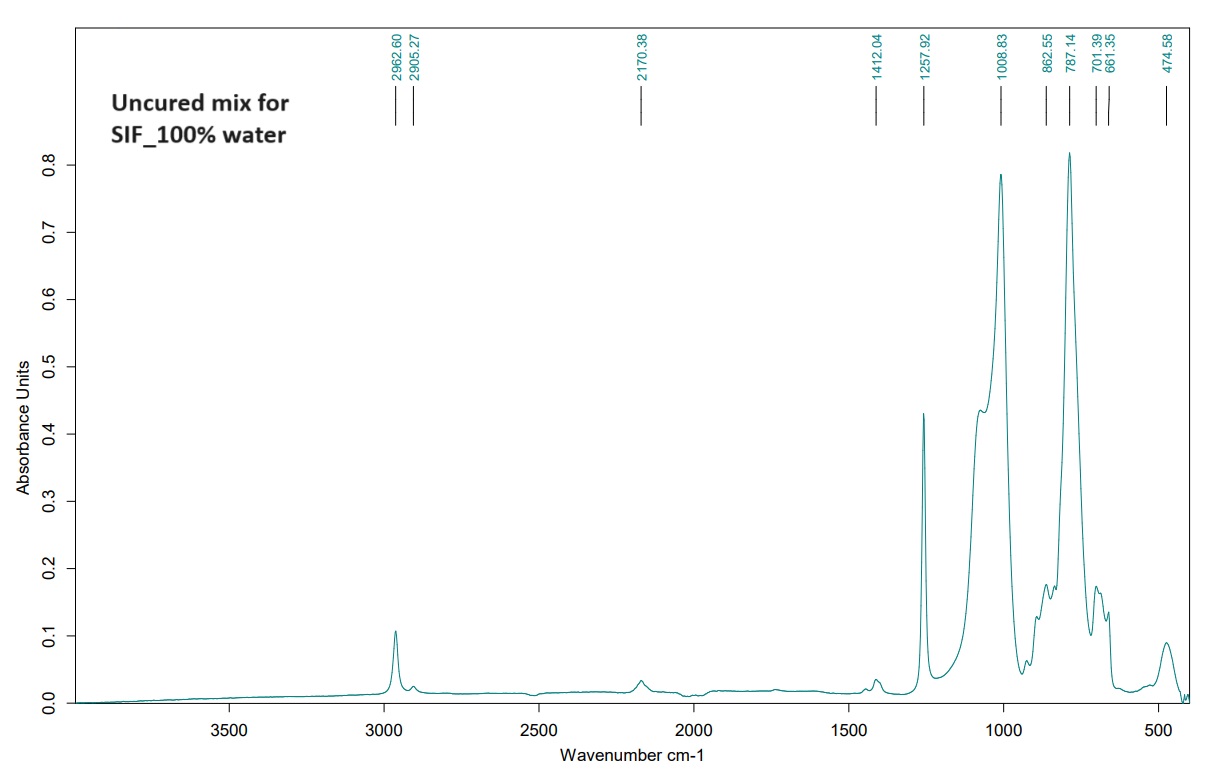

Supplement: Supplementary file 1 [file polymers-15-04425-s001.zip › Figure S2_FTIR_100water uncured.jpg]

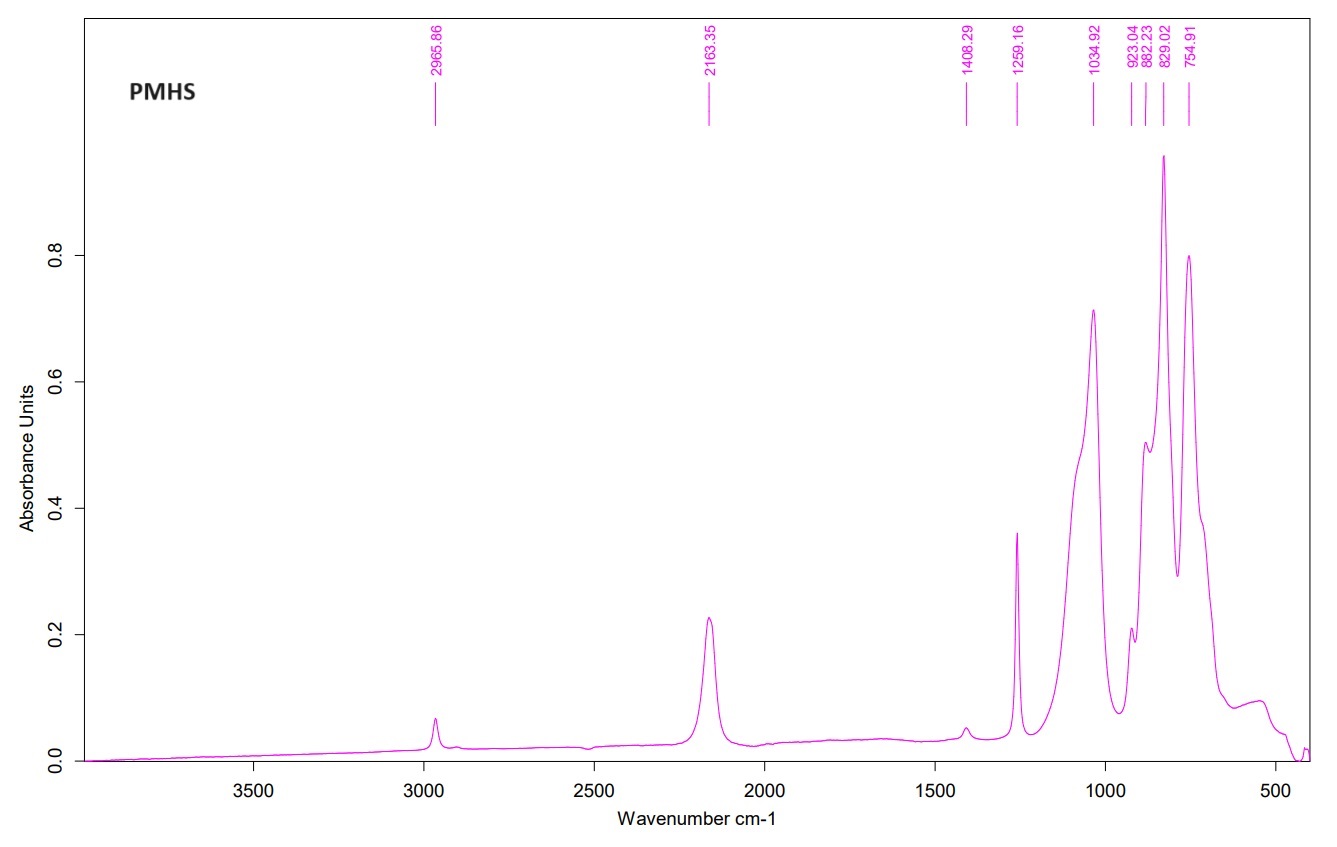

Supplement: Supplementary file 1 [file polymers-15-04425-s001.zip › Figure S3_FTIR_PMHS.jpg]

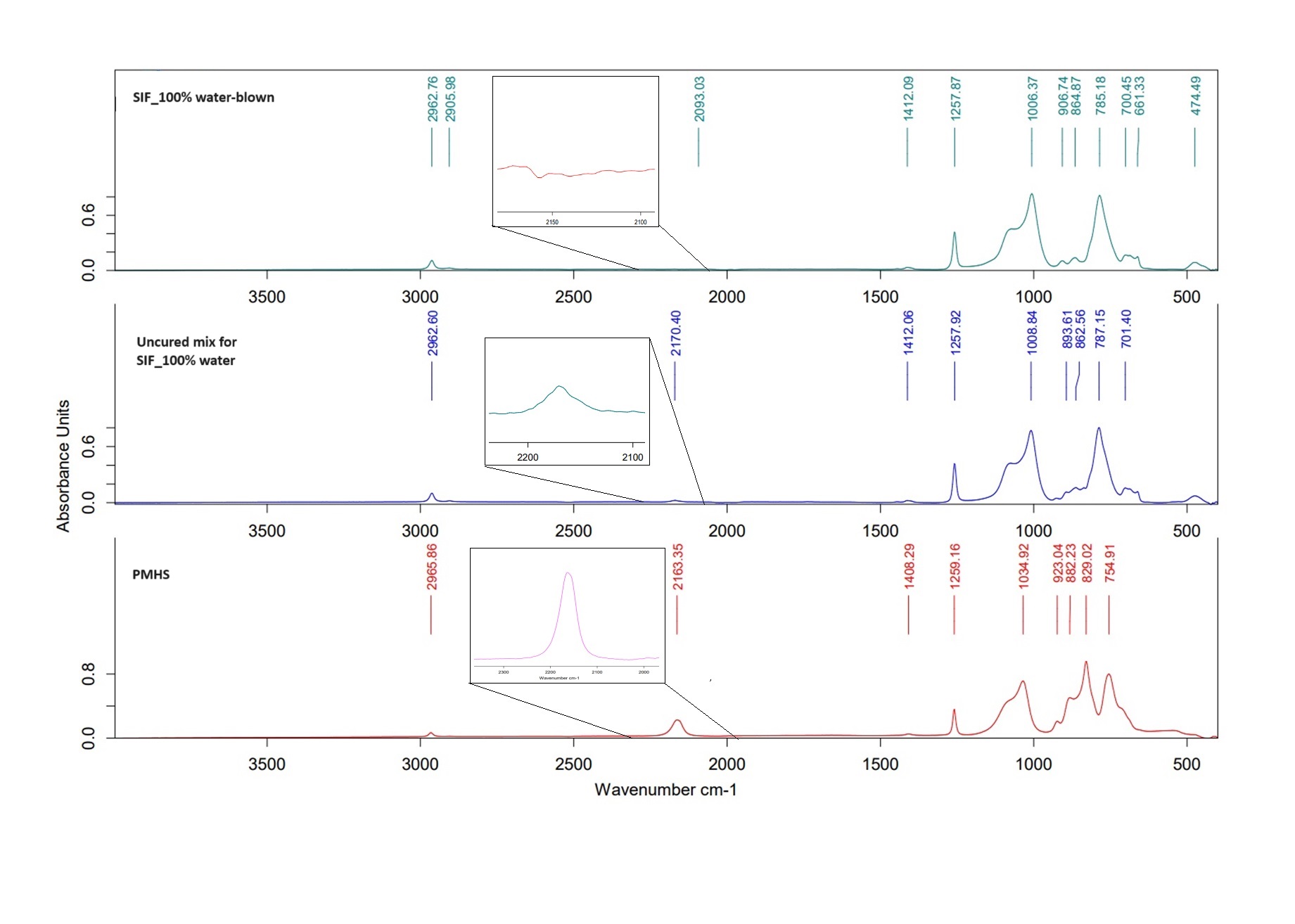

Supplement: Supplementary file 1 [file polymers-15-04425-s001.zip › Figure S4_FTIR_PMHS vs 100w uncured vs 100v cured foam + zoomins.jpg]

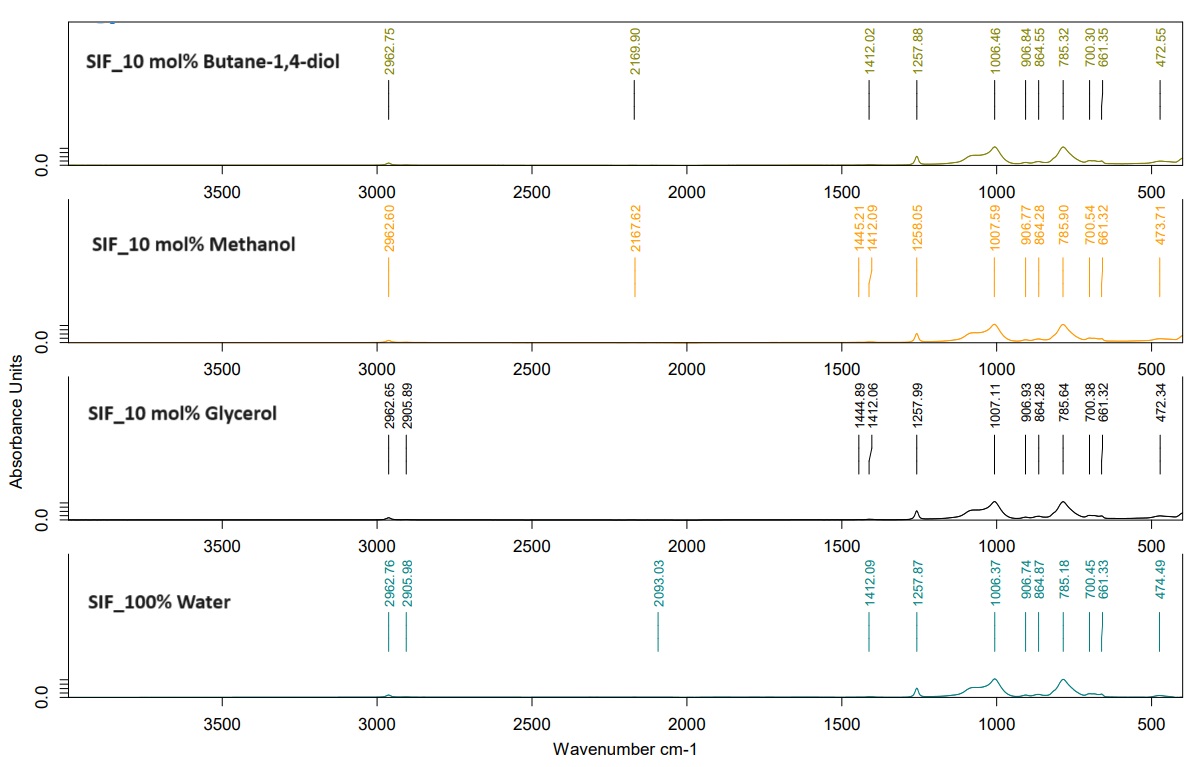

Supplement: Supplementary file 1 [file polymers-15-04425-s001.zip › Figure S5_FTIR_Comparison of SIFs.jpg]
